# Human T Cell Response to Dengue Virus Infection

**DOI:** 10.3389/fimmu.2019.02125

**Published:** 2019-09-04

**Authors:** Yuan Tian, Alba Grifoni, Alessandro Sette, Daniela Weiskopf

**Affiliations:** ^1^Division of Vaccine Discovery, La Jolla Institute for Immunology, La Jolla, CA, United States; ^2^Department of Medicine, University of California, San Diego, La Jolla, CA, United States

**Keywords:** dengue, CD4 T cell, CD8 T cell, T cell epitope, vaccine

## Abstract

DENV is a major public health problem worldwide, thus underlining the overall significance of the proposed Program. The four dengue virus (DENV) serotypes (1–4) cause the most common mosquito-borne viral disease of humans, with 3 billion people at risk for infection and up to 100 million cases each year, most often affecting children. The protective role of T cells during viral infection is well-established. Generally, CD8 T cells can control viral infection through several mechanisms, including direct cytotoxicity, and production of pro-inflammatory cytokines such as IFN-γ and TNF-α. Similarly, CD4 T cells are thought to control viral infection through multiple mechanisms, including enhancement of B and CD8 T cell responses, production of inflammatory and anti-viral cytokines, cytotoxicity, and promotion of memory responses. To probe the phenotype of virus-specific T cells, epitopes derived from viral sequences need to be known. Here we discuss the identification of CD4 and CD8 T cell epitopes derived from DENV and how these epitopes have been used by researchers to interrogate the phenotype and function of DENV-specific T cell populations.

## DENV Infection and the Complex Roles of T Cells

Dengue virus (DENV) belongs to the genus *Flavivirus* and is closely related to several other flaviviruses including Zika virus (ZIKV), yellow fever virus (YFV), Japanese encephalitis virus (JEV), and West Nile virus (WNV) (1). DENV is a serious public health issue especially in tropical and subtropical areas, and it is estimated that ~390 million people are infected yearly with DENV (2). DENV infection is associated with a range of clinical manifestations, from asymptomatic to more severe presentations including dengue hemorrhagic fever (DHF) and dengue shock syndrome (DSS). There is currently no specific therapy available for the treatment of dengue diseases other than supportive care. Furthermore, Dengvaxia® (Sanofi Pasteur), the first licensed DENV vaccine, is associated with efficacy and safety concerns (3–7). Sridhar et al. integratively analyzed data from three clinical trials and reported that Dengvaxia® increases the risk of severe dengue and hospitalization among vaccinees who have not been exposed to DENV before the vaccination (8). In order to develop effective DENV therapeutics and vaccines, it is important to define immunological correlates of protection against DENV infection as well as biomarkers that can be used to access their safety and efficacy.

Although T cells have important functions in combating viral pathogens, both pathological and protective effects of T cells have been reported in the context of DENV infection (9–14). According to T cell original antigenic sin, cross-reactive T cells that are specific for a primary DENV serotype become predominant during a secondary heterologous infection (9–16). Consequently, the expansion of preexisting cross-reactive and low-affinity memory T cells results in ineffective viral control and contributes to immunopathology and severe dengue disease through excessive production of inflammatory cytokines (9–16). In contrast to the implications of original antigenic sin, several lines of evidence indicate that T cells contribute to the control of DENV infection. Murine studies demonstrate that CD4 T cells and especially CD8 T cells can play a protective role against DENV challenge (17–24). Furthermore, HLA alleles associated with protection from severe dengue disease are also associated with strong and multifunctional T cell responses, supporting the notion that T cells have protective functions during DENV infection (25–28). The main characteristic of an efficient vaccine is the prophylactic effect provided by protective neutralizing antibodies. Therefore, it is possible that in Dengvaxia® vaccines, native conserved masked conformational DENV (1–4) epitopes are not unmasked and therefore not accessible for highly neutralizing and broadly protective antibodies. Nevertheless, Dengvaxia® is a yellow fever dengue chimeric vaccine and lacks DENV non-structural (NS) proteins that contain a large proportion of T cell epitopes (25, 28, 29). Therefore, the suboptimal efficacy of Dengvaxia® may partially due to its defective ability to induce T cell responses (30). Indeed, a single dose of the live attenuated tetravalent DENV vaccine TV003 provides complete protection against infection with a DENV-2 challenge virus (31), potentially highlighting the importance of harnessing the protective functions of both humoral and cellular antiviral immunity.

## Metadata Analysis of DENV-Derived CD4 and CD8 T Cell Epitopes

Human antigen-specific T cell immune responses are driven by two factors that are host specific. First the capability of antigen-derived peptides to be bound and presented in the context of HLA class I and II molecules. Second, the immunogenicity of those peptides that depends on the capability of T cells to recognize through T cell receptor (TCR) the HLA-peptide complex and trigger T-cell specific immune responses. Several studies have identified the DENV epitopes able to induce CD8 and/or CD4 T cells specific-response and consecutively the immunodominance of DENV proteins for DENV-specific T cell response. In this review, we summarize previous published data of all the DENV-epitopes experimentally identified by us and others by performing an overall analysis of data available in Immune Epitope Database (www.IEDB.org).

The IEDB database was queried on July 8th 2019 using the following search parameters: Positive assays only, Organism: Dengue virus (ID:12637), No B cell assays, No MHC ligand assays, Host: Homo sapiens (Human). This query retrieved a total of 57 different publications (Table 1). Most of the studies were focused on NS3 protein or multiple DENV proteins defined as immunodominant region based on previous studies (Table 1). Additional studies evaluated the full DENV polyprotein targeting either HLA-Transgenic DENV infected mice (32, 72) or human samples. Specifically, we considered human samples derived from different geographical locations and collected from either healthy DENV seropositive blood donors DENV (25, 26, 28, 73, 77, 80) or patients during acute dengue infection (29, 74, 75) or healthy donor after experimental vaccination with dengue virus (76, 78, 81) (Table 1). A total of 2191 epitopes were described in the 57 references retrieved by the query (Table 1); of those, 825 were restricted by HLA class I molecules, and 1345 epitopes were restricted by HLA class II. To define the most dominant epitopes, we considered epitopes that were reported positive in multiple donors and/or multiple studies. For example, the NS3_1608−1618_ is the most dominant HLA class I epitope, being independently identified by several studies also incorporating different amino acid variants (16, 28, 33–35, 53, 73) (Supplementary Table 1A). Conversely, in the case of HLA class II molecules the C_50−64_ and C_72−86_ epitopes are examples of dominant epitopes, recognized in the majority of donors tested (12 positive out of 17 and 9 positive out of 11, respectively) in several independent studies (25, 77, 78). Supplementary Tables 1A,B list the most dominant class I and class II epitopes. Specifically, to identify the top ~80 epitopes we selected class I epitopes recognized in 5 donors or more (84 epitopes), and class II epitopes recognized in 8 donors of more (77 epitopes).

**Table 1:** List of references for DENV-specific T cell epitopes.

**Protein target**	**#of studies**	**HLA restriction**	**References**
NS3 protein	13	Class I	(16, 32–43)
	7	Class II	(44–49)
	3	Class I and II	(50–52)
Multiple DENV proteins or epitopes	12	Class I	(32, 53–63)
	3	Class II	(64–66)
	5	Class I and II	(67–71)
Polyprotein	6	Class I	(28, 72–76)
	5	Class II	(25, 26, 77–79)
	3	Class I and II	(29, 80, 81)
Total	57		

*DENV epitopes studies divided per protein target and HLA restriction and available in the IEDB (www.IEDB.org)*.

## Immunodominant DENV Proteins Recognized by CD4 and CD8 T Cell

Dissecting the immunodominance of T cell response has considerable implications in vaccine design as the first licensed Dengvaxia® vaccine (Sanofi Pasteur) is composed of DENV structural proteins (prM, E) in a yellow fever backbone while both TV003 (NIH) and TDV (Takeda) vaccines contains both DENV structural and non-structural proteins (31, 82, 83). To assess DENV immunodominance without the bias of adding multiple studies that take in consideration only a single or few DENV proteins, we focused on the 14 studies performed on the full DENV polyprotein and summarized in Table 1.

**Figure 1:**
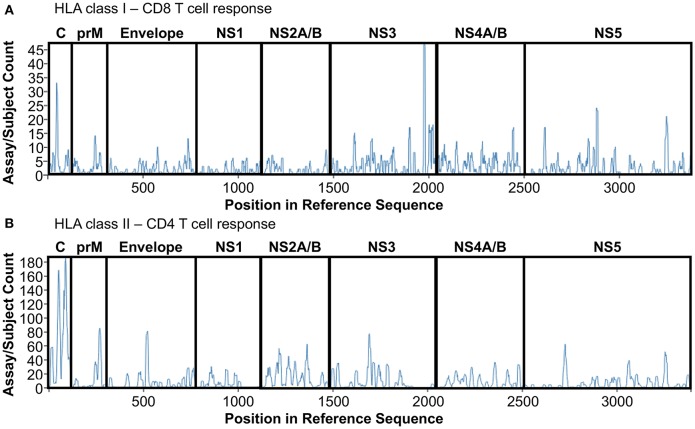
DENV T cell immunodominance. List of epitopes derived from DENV polyprotein studies summarized in Table 1 have been extracted and plotted on a reference DENV sequence (ID:12637) by using Immunobrowser. **(A)** HLA class I restriction. **(B)** HLA class II restriction.

Epitopes derived from those 14 studies have been extracted from IEDB, redundant epitopes independently defined in donors with different HLA restriction have been included to give prominence to promiscuous peptides able to be presented and recognized by different HLA molecules. Finally, epitopes have been divided based on class I or class II restriction and plotted on a DENV reference sequence (ID:12637) by using the Immunobrowser tool (http://tools.iedb.org/immunomebrowser/) (84) freely available on the IEDB. Figure 1 shows the immunodominance of CD8 (Figure 1A, HLA class I restriction) and CD4 T cells (Figure 1B, HLA class II restriction), respectively.

CD8 T cell responses targets mainly NS3 protein, followed by Capsid, NS5 and NS4A/B proteins (Figure 1A). NS3 immunodominance is confirmed throughout all the different studies used for the analysis, representing the most frequent target of CD8 T cell response, disregarding geographical location, and HLA restriction.

Conversely, CD4 T cell responses targets mainly Capisd followed by Envelope, NS3, NS2A/B, and NS5 proteins (Figure 1B). Capsid immunodominance is consistently identified in all the different studies analyzed, representing the most frequent target of CD4 T cell response. Finally, protein immunodominance for both CD4 and CD8 T cells is also function of the multiple exposure of DENV infection, that tends to skew protein immunodominance toward epitopes highly conserved across the different DENV serotypes as previously reported (85). Overall, T cell protein immunodominance is quite complex and widely focused on different protein targets, suggesting that in order to trigger an efficient DENV-specific T cell response both Structural (C, prM, Envelope) and Non-Structural (NS1-5) proteins are required.

## Characterization of Human DENV-Specific T cell Responses

### Megapool Approach to Detect DENV-Specific T Cell Responses

Epitope identification studies have provided the basis for phenotyping DENV-specific T cell responses directly *ex vivo* without the need for *in vitro* stimulation that could potentially alter T cell phenotypes. Utilizing the knowledge of the epitopes recognized we developed the megapool approach, which allows for combining a large number of peptides into one peptide pool based on sequential lyophilization. This enables detection of DENV-specific T cell responses irrespective of HLA types and DENV serotypes in various immunological contexts where only small amounts of blood are available (78). DENV megapools have been generated for both CD4 and CD8 T cells, which consist of 180 and 268 peptides, respectively (25, 27, 28, 73, 77). These peptides are pooled, lyophilized, and resuspended to form a master mix, which is then used to stimulate T cell *ex vivo* (86). DENV CD4 and CD8 megapools account for 62 and 90% of the IFN-γ response in Sri Lankan and Nicaraguan cohorts, respectively, and have been validated in different geographical locations supporting their global applicability (25, 27, 28, 73, 77).

### DENV-Specific CD8 T Cells: Activated, Skin-Homing, and Functional

Using tetramers incorporating three variants of the HLA-A^*^1101-restricted DENV NS3_133−142_ epitope, Friberg et al. reported that cross-reactive CD8 T cells develop following both primary and secondary DENV infections and that the magnitude of tetramer^+^ CD8 T cell response does not correlated with disease severity (35). Although tetramer^+^ CD8 T cells upregulate the activation marker CD38 during the acute phase of infection (35), the phenotype of these DENV-specific CD8 T cells was not further assessed in this study. More recent studies using DENV peptide pools shows that higher magnitude and more polyfunctional CD8 T cell responses correlate with HLA alleles that are associated with reduced risk of severe dengue disease (27, 28), which is consistent with the report that the frequency of DENV-specific cytokine-producing CD8 T cells is higher among children who subsequently developed subclinical secondary infection than those who developed symptomatic secondary infection (87). Using a pool containing 268 CD8 T cell epitopes derived from DENV (termed megapool), de Alwis et al. demonstrated that the majority of DENV-specific IFN-γ^+^ CD8 T cells have a CD45RA^−^CCR7^−^ effector memory (Tem) or CD45RA^+^CCR7^−^ effector memory re-expressing CD45RA (Temra) phenotype (27). Notably, DENV-specific CD8 T cells are also associated with increased PD-1 expression in donors expressing the immunodominant allele HLA-B^*^35:01. In contrast to classical exhausted CD8 T cells, these DENV-specific PD-1^+^ CD8 T cells do not co-express other exhaustion makers and are apparently proliferative and functional (27), suggesting that PD-1 may serve as a marker of activated and highly functional antigen-specific CD8 T cells in the context of DENV infection.

Since DENV infection initiates at the site of the mosquito bite in the host skin, it is possible that CD8 T cells may migrate to the site of infection and mediate localized responses. Indeed, DENV NS3 27-specific CD8 T cells in the periphery blood upregulate the expression of several chemokine receptors including CCR5, CXCR3, and CXCR6 as well as the skin-homing molecule cutaneous lymphocyte-associated antigen (CLA) during acute DENV infection (67). Moreover, DENV-specific CD8 T cells are readily detectable in the skin of DENV-infected individuals at the acute stage (67), suggesting that these cells may exert effector functions at the site of infection. Tissue-resident memory T (Trm) cells reside in non-lymphoid tissues including the skin and can serve as a front line of defense against invading pathogens such as vaccinia virus (88). It would be interesting to investigate whether DENV-specific CD8 T cells could differentiate into Trm cells in the skin that may mount rapid and localized protective immunity upon reinfection.

Comprehensive transcriptomic profiling of DENV-specific CD8 T cells has also been carried out. Chandele et al. performed microarray analysis on HLA-DR^+^CD38^+^ activated CD8 T cells isolated from the PBMCs of DENV-infected patients and found that these cells upregulate genes involved in T cell proliferation, activation, migration and cytotoxicity (89). Interestingly, HLA-DR^+^CD38^+^ CD8 T cells also display increased expression of multiple inhibitory receptors and downregulate several genes that are involved in TCR signaling (89). A more recent study from our group characterized the transcriptomic profiles of DENV-specific CD8 Tem and Temra subsets identified by their production of IFN-γ following simulation with the megapool of DENV-derived epitopes (90). DENV-specific Tem and especially Temra cells display specialized gene expression profiles and upregulated genes that are associated with activation, co-stimulation, and effector functions (90), which is consistent with the previous study from Chandele et al. Since these DENV-specific Tem and Temra cells were isolated from healthy donors with secondary DENV infection, these studies suggest that DENV-specific CD8 T cell populations may maintain an activated phenotype in donors that have been infected multiple times with DENV. Interestingly, DENV-specific Temra cells may have higher expression of a few killer cell immunoglobulin-like receptor (KIR) genes including *KIR2DL3* by comparison with DENV-specific Tem cells (90). In addition, DENV-specific CD8 T cells may show preferential usage of TCR beta-chain variable (TRBV) genes (90), which is in line with the report that DENV NS3_133_-specific CD8 T cells targeting HLA-A^*^11:01-restricted epitope variants derived from DENV1, DENV3, and DENV4 but not DENV2 preferentially use a few TRBV segments including TRBV9, TRBV12-3/4, and especially TRBV11-2 (33).

### DENV-Specific CD4 T Cells: A Tale of Cytotoxicity

The majority of antigen-specific CD4 T cells differentiate into T helper type 1 (Th1) and follicular helper T (Tfh) cells following viral infections and provide help to CD8 T cells and B cells (91–93). Indeed, DENV-specific CD4 T cells produce Th1 cell-associated cytokines including IFN-γ, TNF-α, and IL-2 following both infection and vaccination (87, 94, 95). In addition, DENV-specific CD4 T cells with cytotoxic activity have been reported by numerous studies (12) and their frequency may be lower in patients with more severe dengue disease (96). Interestingly, a subset human CD4 T cells, which is CD45RA^+^CCR7^−^ and termed effector memory re-expressing CD45RA T (Temra) cells, expands in individuals that have been infected with DENV multiple times, and the frequency of DENV-specific CD4 Temra cells is higher in donors expressing an HLA allele associated with protection from severe dengue disease (26). Despite the production of IFN-γ, these cells may not represent classical Th1 cells as they lack the expression of CXCR3 (26). CD4 Temra cells have increased expression of several cytotoxic molecules including CD107a, perforin, granzyme B as well as the CX3CL1 (fractalkine) receptor, CX3CR1 (26). Notably, CX3CR1 has recently been reported to be a member of a 20-gene set that can predict severe dengue disease (97). Subsequent transcriptomic profiling studies further revealed the gene expression patterns and heterogeneity of CD4 Temra cells and identified additional phenotypic markers such as GPR56 and CD244 that are specifically expressed by cytotoxic CD4 Temra cells and confirmed their expression on DENV-specific Temra cells (98, 99). Additionally, cytotoxic CD4 Temra cells may have undergone extensive clonal expansions based upon TCR analysis (98, 99), supporting the notion that these cells are induced by repeated DENV infections.

Both Foxp3^+^ regulatory T (Treg) cells and Foxp3^−^ type 1 regulatory T (Tr1) cells can suppress inflammation and exert immunoregulatory effects (100, 101). However, their functional significance in the context of DENV infection is less well-defined (14). It has been reported that the frequency of Treg cells and the ratio of Treg cells to effector T cells are significantly higher during acute DENV infection than after recovery in patients with mild disease but not in those with severe disease (102). However, subsequent studies indicate that the frequency of Treg cells is not associated with viral load or disease severity (103). Therefore, whether and how Treg and Tr1 cells influence antiviral immune response and disease progression during DENV infection warrants further investigation.

T cells help to B cells is provided by a CD4 T cells subset termed follicular T helper cells (Tfh) (104). Tfh cells have been associated with protective roles in human infectious disease (105–107) and vaccinees (108–110). They provide several forms of T cell help to B cells such as signals that promote survival, proliferation, plasma cell differentiation, hypermutation, class-switch recombination, adhesion and chemoattraction (cell migration) (111). Tfh cells are essential for the generation of most isotype switched and affinity matured antibodies, and therefore they have an obvious role in protective immunity against pathogens. A recent breakthrough has been the ability to detect Tfh cells in peripheral blood (105, 112) thus allowing their assessment in PBMC samples based on surface markers. A central marker of Tfh cells is the CXC-chemokine receptor 5 (CXCR5) which is required for T and B cells to enter into follicles. OX40, and PD-L1 have further been identified as TCR activation-dependent markers of human Tfh cells (113, 114). Recent studies have reported an expansion of peripheral Tfh cells in DENV-infected children during the acute phase (115). Furthermore, Tfh cells are more abundant in patients with secondary DENV infection and in those who developed a more severe dengue disease (115). Although Tfh cells has been shown to promote DENV-specific antibody responses in mice (116), the differentiation and functional significance of DENV-specific Tfh cells in humans warrants further investigation.

## Conclusion and Perspective

**Figure 2:**
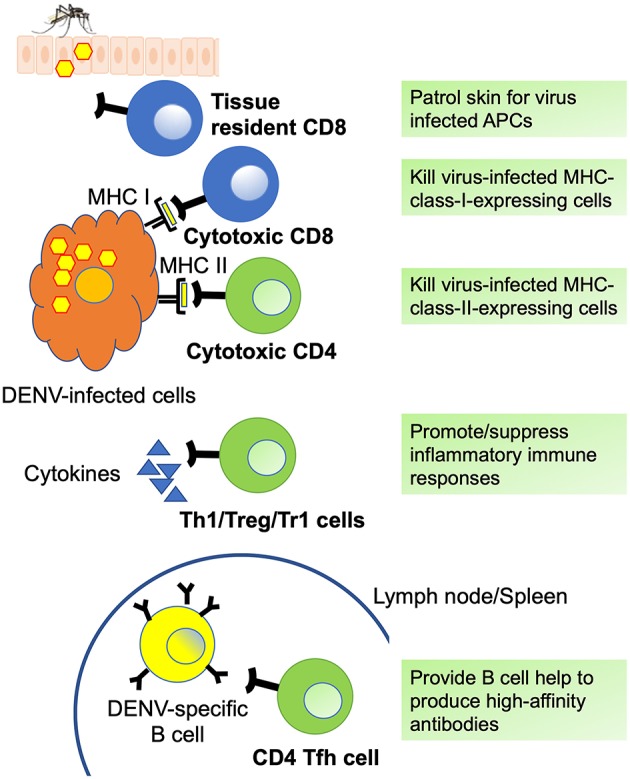
DENV specific T cell phenotypes. Summary of DENV-specific CD8 (blue) and CD4 (green) T cell phenotypes and functions. Tissue resident CD8 T cells patrol skin for infected antigen presenting cells (APCs) and can generate immediate effector functions. Cytotoxic CD8 and CD4 T cells express cytotoxic molecules such as granzyme B and perforin and can kill virus-infected cells via MHC I- and MHC II-dependent mechanisms. Th1 cells mediate and promote antiviral immune responses via the production of inflammatory cytokines such as IFN-γ and TNF-α, whereas regulatory CD4 T cells including Treg and Tr1 cells suppress inflammatory immune responses by producing cytokines such as IL-10 and TGF-β. Tfh cells provide help to DENV-specific germinal center B cells (yellow) and are essential for optimal germinal center reactions, thus promoting the generation of high-affinity antibodies, memory B cells, and long-lived plasma cells.

All DENV-specific T cell phenotypes discussed in this review as well as the markers they express are summarized in Figure 2 and Table 2, respectively. Comprehensive epitope identification over the last few years has provided the tools that allow one to probe for DENV specific T cell responses in donors exposed to natural infection and vaccination. Global assessment of dengue virus-specific CD4 and CD8 T cell responses in dengue-endemic areas led to the development of new tools (megapools) that allow analysis of small samples typically available from pediatric and hospital cohorts. It has also been demonstrated that DENV-specific CD4 and CD8 responses are more complex than previously thought, with different subsets revealed by in depth phenotypic and transcriptomic analyses. The hypothesis that these different subsets have unique roles and dictate and shape clinical outcomes and vaccine efficacy will have to be explored in the near future.

**Table 2:** Markers expressed by DENV-specific T cell populations.

**T cell population**	**Makers**
Skin-homing CD8 T cells	CCR5, CXCR3, CXCR6, CLA
CD4/CD8 Tem	CD45RA^−^CCR7^−^
CD4/CD8 Temra	CD45RA^+^CCR7^−^
Cytotoxic CD8 T cells	Perforin, Granzyme B, PD-1
Cytotoxic CD4 T cells	Perforin, Granzyme B, CX3CR1, GPR56, CD244
Th1 cells	IFN-γ, TNF-α, and IL-2
Tfh cells	CXCR5, PD-1, OX40, PD-L1

## Author Contributions

All authors listed have made a substantial, direct and intellectual contribution to the work, and approved it for publication.

### Conflict of Interest Statement

The authors declare that the research was conducted in the absence of any commercial or financial relationships that could be construed as a potential conflict of interest.
